# Feasibility of the Big 5—Jena eCS Protocol: First Experience Implementing a New Extended CT Protocol in the Initial Diagnostics of Ischemic Stroke

**DOI:** 10.1007/s00062-021-01058-6

**Published:** 2021-08-11

**Authors:** Moisés F. Molina-Fuentes, Rotraud Neumann, Wilhelm Behringer, Marcus Franz, P. Christian Schulze, Otto W. Witte, Albrecht Günther, Carsten Klingner, Lukas Lehmkuhl, Beatrice Steiniger, Ulf Teichgräber, J. E. Rod, Thomas E. Mayer

**Affiliations:** 1 Department of Neuroradiology, Jena University Hospital—Friedrich Schiller University, Jena, Germany; 2 Department of Diagnostic and Interventional Radiology, Medical Center—University of Freiburg, Faculty of Medicine, University of Freiburg, Freiburg, Germany; 3 Institute for Diagnostic and Interventional Radiology, Jena University Hospital—Friedrich Schiller University, Jena, Germany; 4 Emergency Department, Jena University Hospital—Friedrich Schiller University, Jena, Germany; 5 Department of Internal Medicine, Jena University Hospital—Friedrich Schiller University, Jena, Germany; 6 Hans Berger Department of Neurology, Jena University Hospital—Friedrich Schiller University, Jena, Germany; 7 Clinic for Radiology, Rhön-Klinikum Campus Bad Neustadt, Bad Neustadt a. d. Saale, Germany; 8 Institute of Health and Biomedical Innovation, Queensland University of Technology, Brisbane, Australia

**Keywords:** Cerebrovascular, Ischemia, Tomography, Thromboembolic, Ultrasound, Cardiovascular thrombi, Cardiac ultrasound

## Abstract

**Purpose:**

The most common protocols in the initial diagnostic of acute ischemic stroke do not assess cardiogenic or aortic causes of embolism. These are usually evaluated later by transthoracic (TTE) or transesophageal (TEE) echocardiography. This study aimed to evaluate the feasibility of a diagnostic tool for thoracic cardiovascular thrombi according to the first experience with a new extended cardio-stroke protocol (Big 5—Jena eCS protocol) in acute stroke patients.

**Methods:**

Retrospective analyses of the tomography scans database of the Jena University Hospital were performed. We included a total of 67 patients in the feasibility analyses, based on the evaluation of three outcomes.

**Results:**

Primary outcome: the Big 5—Jena eCS protocol was able to detect thoracic cardiovascular thrombi in a total of 20 patients in different locations including the arch of the aorta, the aortic valve, the left atrium, the left atrial appendage, the left ventricle, and the pulmonary arteries. Secondary outcome: implementating the protocol did not result in a significant elevation of the radiation exposure compared to traditional protocols. Tertiary outcome: the new protocol identified seven cases that were considered negative by echocardiography.

**Conclusion:**

The implementation of an extended cardio-stroke protocol is feasible, no significantly time-consuming, acquiring assessable imaging, and maintaining radiation exposure acceptable. The Big 5—Jena eCS protocol was also able to detect some thrombi not reported by TTE or TEE; however, due to our data’s explorative character, a conclusive comparison with cardiac ultrasound is not possible. A prospective pilot study and clinical trials should be conducted to assess the diagnostic accuracy of this protocol compared to echocardiography and determine the potential impact on diagnostic and treatment decisions.

## Introduction

Stroke is one of the most common diagnoses seen in the emergency department and contributes to significant morbidity and mortality in the adult population [1–4]. A substantial number of stroke patients are often affected by underlying heart conditions leading to an ischemic event [4, 5]. About 80–85% of strokes are ischemic events caused by a cerebral artery’s acute occlusion [6–8]. Based on the TOAST criteria of suspected etiology (Trial of ORG 10172 in Acute Stroke Treatment), ischemic strokes can be classified as cardioembolic, large artery atherosclerosis, small vessel disease, other known etiology, or undetermined etiologies [5, 9]. Cardiogenic embolism is responsible for about 20–40% of large vessel occlusion in stroke patients [2, 7, 10, 11].

The examination of the brain through a non-contrast brain computed tomography (CT), perfusion tomography (CT perfusion) of the brain, and CT angiography (CTA) of the cerebral arteries are commonly used protocols in the diagnosis of an acute ischemic stroke [12, 13]. Typical localization sites of cardiac thrombi such as the left atrium (LA), the left atrial appendage (LAA), or the aortic valve (AV) are not detected in those examinations [5, 14]. Transthoracic echocardiography (TTE) as a non-invasive method and transesophageal echocardiography (TEE) as a semi-invasive examination are the clinical standard for detection of cardiac thrombi [10, 11, 15]; however, the implementation of these examinations requires a clinically stable patient and is time-consuming. Therefore, these examination techniques are not indicated in the context of an acute stroke.

Cardiac CT might be a reliable alternative, even in the context of acute ischemic stroke in the emergency department (ED) [16–18]. By avoiding performing traditional (semi) invasive examinations for diagnosing cardiac thrombi, the complications associated with these methods can also be abolished. Furthermore, the additional information about thrombotic findings in the heart and aorta as soon as the patient arrives in the ED could potentially have an impact in early stroke treatment [1, 3, 10]. Therefore, the present study aimed to evaluate the feasibility of a diagnostic tool for cardiogenic and vascular thrombi based on the experience with a new extended cardio-stroke protocol (Big 5—Jena eCS protocol) in acute stroke patients. Thus, the study ambition was not related to prove diagnostic accuracy or to test processes that future clinical trials could use in order to prove diagnostic accuracy (the methodology of a pilot study). In the present study the main question was to evaluate whether a radiological test works. In this scenario, the most recent literature suggests that feasibility studies are the appropriate methodological design [19].

## Material and Methods

### Big 5—Jena eCS Protocol Design

At the Institute for Diagnostic and Interventional Radiology (IDIR) of the Jena University Hospital (Universitätsklinikum Jena [UKJ]) in Thuringia, Germany, we designed an electrocardiogram (ECG) gated CT protocol combining diagnostics for acute ischemic stroke including brain perfusion, cerebral arteries, and thoracic cardiovascular thrombi (extended cardio-stroke protocol, Big 5—Jena eCS protocol).

This protocol is performed using a multi-slice (256 detector row) GE Revolution CT Scanner (General Electric Healthcare, Chicago, IL, USA), and 110 ml of contrast medium Iomeprol 400 mg iodine/ml (Imeron® 400 MCT Bracco Imaging, Milan, Italy). As a result, we can obtain the following in a single examination:

1. A non-enhanced brain CT.
2. A CTA involving a combination of an ECG-triggered axial scan over the heart and the arch of aorta followed by a helical scan without triggering up to the brain. Contrast medium (CM) application: 60 ml (4 ml/s).
3. A venous axial scan of the LAA.
4. A CT perfusion of the brain. CM application: 50 ml (4 ml/s).

Compared with the standard imaging in stroke patients, our new protocol increases the intravenous contrast to 110 ml, which means only a 10 ml addition.

**Fig. 1:**
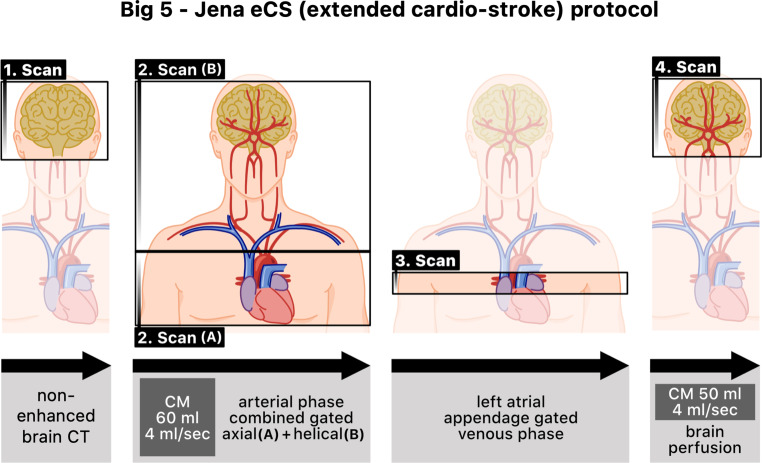
Novel extended CT protocol in the initial ischemic stroke diagnostics. *CT* computed tomography, *CM* contrast medium

The ECG triggering is made using a cardiac profile depending on the heart rate. The bolus tracking is performed by defining a threshold of 110 Hounsfield units (HU) in the ascending aorta with a delay of 6 s. The scan time for a 180° scan over the heart is 140ms. A schema of our new Big 5—Jena eCS protocol is presented in Fig. 1.

With this protocol, we can evaluate five essential aspects in the context of stroke. Firstly, we can assess the cerebral tissue, the cerebral perfusion, and the intracranial arteries responsible for the brain’s blood supply. We can also evaluate the arch of aorta (AA), the vertebral arteries (VA), and the carotid arteries. Furthermore, we also visualize the heart structures, including the left ventricle (LV), the aortic valve (AV), the LA, and the LAA.

### Sample

**Fig. 2:**
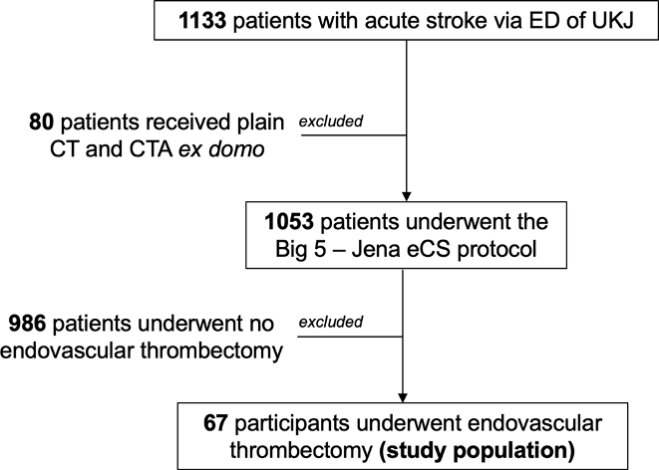
Flow diagram of the study sample and diagnostic methods to evaluate cardiovascular thrombi. *CT* computer tomography, *CTA* computed tomography angiography, *eCS* extended cardio-stroke, *ED* emergency department, *UKJ* Universitätsklinikum Jena (Jena University Hospital)

Overall, 1133 consecutive patients with acute stroke symptoms were admitted in the ED of the UKJ and 1053 patients were assessed with the new Big 5—Jena eCS protocol. This number was reached based on the exclusion of 80 patients who underwent a previous non-enhanced brain CT and CTA before being transferred to the ED of the UKJ. We included a subsample of patients that underwent endovascular thrombectomy to conduct the analyses, as these patients were more likely to have underlying thoracic cardiovascular thrombi relative to those on who no endovascular thrombectomy was performed. This procedure yielded a total of 67 participants after excluding 986 patients where no endovascular thrombectomy was performed (see Fig. 2).

### Data Analysis

The research team reviewed first the scans obtained by implementing the Big 5—Jena eCS protocol and then proceeded to analyze the echocardiographic reports. In the case of the new protocol, three assessors, a second-year medical resident, and two experienced board-certified radiologists of our ND, one with vast experience in cardiovascular radiology and the other in neuroradiology, independently reviewed post hoc each of the 67 CT scans. Assessors were blinded to the echocardiography results, and any conflicts were discussed until the three assessors reached a consensus.

In the case of the echocardiographic data, examination was performed at a median of 9 days after hospital admission (range 7–30 days). Of the 67 patients 49 underwent echocardiography to identify a potential thrombotic source for the ischemic stroke. We did not perform a systematic secondary analysis and relied on the original data as available from non-contrast echocardiographic assessments of patients performed at the treating physicians’ discretion without systematic secondary analysis by a core laboratory. A TTE was performed in 22 patients and TEE in 27. Echocardiography was not performed in the remaining patients, in 1 case due to early transfer to another hospital, in 3 patients due to the prior evidence of cardiovascular thrombi in the CT scan, in 4 cases due to treating physician’s judgement and in 10 patients due to death after hospital admission.

### Study Outcomes

Based on this information the research team evaluated three outcomes. The primary outcome evaluated the Big 5—Jena eCS protocol’s ability to detect thoracic cardiovascular thrombi. The secondary outcome evaluated the mean dose length product (DLP) as an index for radiation exposure and overall safety. The tertiary outcome devolved on the possibility of the protocol to detect thrombotic deposits not reported by echocardiography.

## Results

A total of 67 patient were included in the feasibility analyses. The general characteristics of the sample of participants are shown in Table 1. There were 37 women (55.2%) and 30 men (44.8%), with a mean age of 70 years ± 11 years (range 46–87 years). Forty patients had atrial fibrillation (AF), and one patient had atrial flutter. Almost half of the patients with AF, i.e., 19 cases, were new-onset diagnoses.

**Table 1:** Demographic and coexisting conditions of the study population and patients with CT detected cardiovascular thrombi

Characteristic	Patients with cardiovascular thrombi (*n* = 20)	Study population (*n* = 67)
Mean age (range) – years	71 (49–87)	70 (46–87)
Gender – *n* (%)		
Male	8 (40)	30 (44.8)
Female	12 (60)	37 (55.2)
Coexisting conditions – *n* (%)		
Atrial fibrillation	13 (65)	40 (59.7)
New onset	9 (45)	19 (28.4)
Previously known	4 (20)	21 (31.3)
Medicated with (med.)	1 (25)	9 (42.9)
Edoxaban	–	4
Apixaban	1	2
Falithrom	–	2
Dabigatran	–	1
Non-medicated	3 (75)	12 (57.1)
Atrial flutter, med. apixaban	–	1 (1.5)
Arterial hypertension	12 (60)	44 (65.7)
Diabetes mellitus	5 (25)	13 (19.4)
Nicotine abuse	2 (10)	13 (19.4)
Heart failure	1 (5)	9 (13.4)
Dyslipidemia	1 (5)	8 (11.9)
Previous myocardial infarction	3 (15)	8 (11.9)
Chronic obstructive pulmonary disease	2 (10)	7 (10.4)
Coronary artery disease	4 (20)	7 (10.4)
Peripheral arterial occlusive disease	1 (5)	5 (7.5)
Renal failure	2 (10)	5 (7.5)
Alcohol abuse	–	4 (6)
Hypothyroidism	–	4 (6)
Osteoporosis	3 (15)	4 (6)
Hyperthyroidism	–	3 (4.5)
Overweight	–	3 (4.5)
Pulmonary hypertension	–	3 (4.5)
Liver cirrhosis	–	3 (4.5)
Persistent foramen ovale	–	2 (3)
Exitus letalis	2 (10)	10 (14.9)

### Primary Outcome

**Fig. 3:**
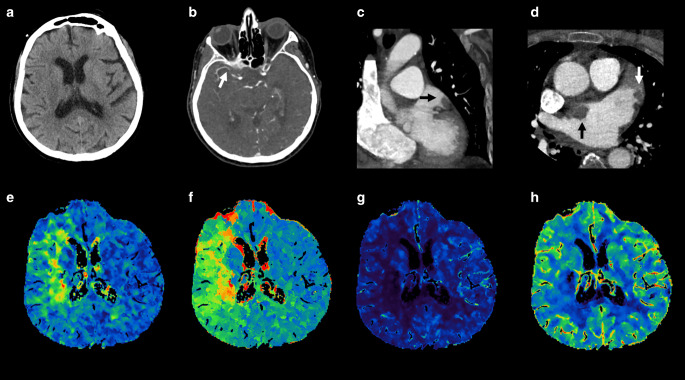
Imaging of a 73-year-old female treated endovascularly at our hospital after initial admission with suspicion of a right hemispheric stroke, occlusion of the middle cerebral artery’s first segment, and simultaneously thrombi in the left atrium and left atrial appendage. **a** Non-enhanced brain computed tomography (*CT*) without any stroke signs, **b** CT angiography showing thrombosis of the right middle cerebral artery’s first segment (*white arrow*), **c** coronal, and **d** axial heart scans show a thrombus in the left atrium (*black arrow*) and left atrial appendage (*white arrow*). The perfusion parameters show **e** increased mean transit time and **f** time to peak with no significant alteration of **g** the cerebral blood flow and **h** the cerebral blood volume in the right middle cerebral artery’s perfusion territory

In CT scans performed by following the Big 5—Jena eCS protocol, thoracic cardiovascular thrombotic findings were observed in 20 patients (29.9%). The primary localization of those thrombi was the AV, which was affected in eight patients, followed by the AA in six individuals and the LAA in five. Furthermore, thrombi were also observed in the LA of four patients, the LV in two patients, and one patient was diagnosed with a pulmonary embolism. An overview of the blood vessel occlusions (carotids, vertebral arteries, and intracranial circulation) associated with the thrombi’s localization in the heart, the aorta, and the pulmonary arteries (PA), including the overlaps, is given in Table 2. Of the 41 patients with cardiac arrhythmias (40 with AF and one with atrial flutter, see Table 1), 13 patients (31.7%) showed thoracic cardiovascular thrombotic findings in CT as follows: two in the LAA, three in the LAA + LA, one in the LA, three on the AV, one on the AV + AA, two in the AA, and one in the LV + PA. For example, Fig. 3 shows the case of a patient admitted to the ED of UKJ in which the Big 5—Jena eCS protocol was implemented. There we can see that thrombi in the LAA and LA were identified, along with a right M1 occlusion.

**Table 2:** Patients with thrombotic findings: thrombi localizations and occluded blood vessels

Intracranial vessel occlusion		Thrombus location in the heart cavities, aorta, and PA							
		LAA	LAA + LA	LA	AV	AV + AA	AA	LV	LV + PA
	*n*	*2*	*3*	*1*	*6*	*2*	*4*	*1*	*1*
*Right ICA*	*1*	–	1	–	–	–	–	–	–
*Left ICA*	*1*	–	–	–	–	–	1	–	–
*Left ICA* *+* *M1*	*1*	–	–	–	1	–	–	–	–
*Left M1*	*2*	–	–	–	1	–	–	–	1
*Right M1*	*10*	2	2	1	2	–	3	–	–
*Left M2*	*2*	–	–	–	1	–	–	1	–
*Left P1*	*1*	–	–	–	1	–	–	–	–
*Right P2*	*1*	–	–	–	–	1	–	–	–
*BA* *+* *left VA*	*1*	–	–	–	–	1	–	–	–

*n* number of patients with each type of vessel occlusion or thrombi localization, *AA* arch of aorta, *AV* aortic valve, *LA* left atrium, *LAA* left atrial appendage, *LV* left ventricle, *PA* pulmonary arteries, *BA* basilar artery, *ICA* internal carotid artery, *M1* middle cerebral artery’s first segment, *M2* middle cerebral artery’s second segment, *P1* posterior cerebral artery’s first segment, *P2* posterior cerebral artery’s second segment, *VA* vertebral artery

### Secondary Outcome

The extended cardio-stroke protocol also leads to slightly higher radiation exposure. In our group of 20 patients with thoracic cardiovascular thrombotic findings, the mean dose length product (DLP) was 2072.5 mGy*cm with a standard deviation (SD) of 360.3 mGy*cm. In a group of 100 patients examined during the same period with a non-enhanced brain CT, CT perfusion, and CTA, the mean DLP was 2038.8 mGy*cm with an SD of 236.1 mGy*cm.

### Tertiary Outcome

Of the 67 participants, the Big 5—Jena eCS protocol detected thoracic cardiovascular thrombi in 20 individuals. Among the 20 individuals in which the CT was positive, only 12 cardiac ultrasounds were performed. This was due to the fact that one patient was transferred to another hospital and two patients died before cardiac echocardiography could be performed; additionally, two patients did not undergo echocardiography based on the treating physician’s judgement and three patients due to the prior evidence of cardiovascular thrombi in the CT scan.

Of the 20 patients with CT-detected cardiovascular thrombotic findings, 4 underwent a TTE and 8 a TEE (40% of cases with suspected thrombi based on CT findings). The TTE was positive in two patients: one patient with thrombus in the LV and one in the LA and negative in two cases with CT-diagnosed thrombi: one with thrombus in the LAA and another with aggregations on the AV. The TEE was positive in three cases: one with thrombi in the LAA, one with thrombi in the LV and a pulmonary embolism, and another on the AV, in which TEE detected spontaneous echocardiographic contrast and negative in five patients: one with affectation of the AA, two with aggregations on the AV, and two patients with simultaneous affectation of the AV and AA. As expected, due to the methodological limitations, the compromise of the AA could not be diagnosed through TTE. Thus, there was no sufficient echocardiographic confirmation in cases with suspected thrombi in CT.

## Discussion

There are already studies comparing echocardiography and cardiac CT for cardiovascular cause finding in stroke patients, but not in the context of acute stroke [1, 4, 8, 10, 11, 16, 18, 20]. We have developed a CT protocol for this purpose, and our initial experience with it has shown that such an approach is possible: the resulting imaging is of sufficient quality, acquisition times are not significantly longer than usual, the incrementation of radiation exposure is minimal, and the contrast medium application was slightly higher compared to the most common protocols. Finally, the protocol was also able to find some important radiological findings that echocardiography might have missed.

The non-systematically quantified time required to set up the ECG triggering was less than 5 min, which was acceptable; however, in recent months, to reduce direct contact with patients whose coronavirus disease 2019 (COVID-19) status is unknown, the ECG triggering was eliminated without significantly affecting image quality. At the same time, the cardiac window was widened, and currently, the entire lung field is being acquired to assess possible pulmonary infiltrates that could indicate a COVID-19 infection.

Regarding the DLP findings, technical advances regarding CT acquisition images, including the iterative reconstruction, have considerably reduced the radiation exposure [3, 4, 21]. The extended cardio-stroke protocol leads to slightly higher radiation exposure than the usual CT protocols [22–25]; however, due to the equivalence of CT and TEE described in the literature regarding thrombus detection in patients after a stroke [5, 15, 17] and the additional possibility of evaluation of the aortic arch, the Big 5—Jena eCS protocol has been used as clinical standard at our radiology institute in the initial assessment of patients with acute stroke and suspicion of an ischemic etiology.

Early phase CT images have great sensitivity in the detection of thrombi [26]. Still, its specificity is suboptimal because of its inability to characterize an apparent filling defect differentiating it between blood stasis and an actual thrombus, leading to easy misdiagnoses of LAA thrombi [1, 10, 27]. In an attempt to avoid this pitfall, we implemented in our study protocol two contrast phases: the initial CTA, which includes the LAA in an early arterial phase, and a late venous phase confined just to the LAA, which allows a homogeneous enhancement of this cavity [1, 24]. Compared to the echocardiography, the CT offers a superior anatomical visualization of the heart structures, allowing the evaluation of LAA and LA enlargement. Noteworthy, circulatory stasis, which is often detected before an LAA thrombus manifests, and LAA/LA enlargement are considered markers of embolism and predictors for stroke recurrence, even independent of atrial fibrillation [4, 28, 29].

Due to its safety and wide availability, the TEE is well implemented in the clinical routine for evaluating stroke patients [10, 30, 31]. Additionally, the TEE is a semi-invasive, time-consuming technique that requires specially trained and skilled healthcare staff to be accurately performed and interpreted [30–32]. Nevertheless, TEE is only sensitive enough to adequately evaluate the LAA, LA, and AV. On the other hand, the TTE is a non-invasive procedure and is not associated with the TEE complications, besides being more sensitive for thrombi in the LV; however, it should be noted that methodologically, it is impossible to evaluate the AA by TTE [28–30, 33].

This single center study is retrospective, descriptive, and therefore bounded by some limitations. A major one is the very explorative character of the data. Additionally, no medication was administered to decelerate the heart rate according to the acute stroke setting. Despite ECG gating, motion artifacts because of normal or even increased heart rate could not be wholly avoided. Therefore, rapid heart rate and motion artifacts can lead to misdiagnosis of small thrombi in the coronary arteries; however, this is not a primary aspect to assess with the Big 5—Jena eCS protocol. Another limitation regarding the echocardiographic report is their operator-dependent nature; thus, our echocardiographers’ experience might affect the results we obtained from the reports. As mentioned, echocardiography was performed days after hospital admission. As part of the medical and endovascular treatment, the thrombolytics administration might have dissolved blood clots when the cardiac ultrasound took place. The anatomical method-related limitations and the therapeutic effects might contribute to the so-called missing findings in the TTE and TEE.

Irrespective of the abovementioned limitations, the cardiac imaging included in our extended cardio-stroke protocol also allows the simultaneous evaluation of atherosclerotic disease as another embolism substrate [3, 4, 34, 35], which could have important therapeutic implications in embolic stroke associated with atrial fibrillation [36–38]. The non-invasive character of CT combined with its reproducibility and the other advantages mentioned above highlight this technique as a reliable option in the assessment of acute stroke patients in the ED [8, 25]. Furthermore, it is possible that the presence of an intracavitary thrombus could support the anticoagulation despite prior fibrinolysis for selected patients with a small or moderate sized brain infarction and no evidence of brain bleeding in the CT scan. Diagnosing the cardiac thrombus simultaneously with the stroke may support early anticoagulation in patients with a high risk for short-term recurrent stroke. A study in Lombardy (Italy) suggested that patients with atrial fibrillation treated with early anticoagulation after an acute stroke had a lower incidence of clinically symptomatic intracranial hemorrhage and that anticoagulation < 48 h was associated with fewer ischemic recurrences in selected patients, without increasing the risk of intracerebral bleeding [36, 39].

## Conclusion

In our first experience implementing the new extended cardio-stroke protocol (Big 5—Jena eCS protocol) regularly, we could establish the protocol’s feasibility in the context of acute stroke. In addition, the new protocol allowed the rapid acquisition of adequate imaging without significantly incrementing the radiation exposure or the contrast medium administration.

Due to the explorative nature of the present feasibility study, we consider its value lies in the generation of a hypothesis that should be confirmed with more robust methodologies in the future. Even though we did not formally evaluate the methodological procedures of the present research in order to reach the status of a pilot study, we think it is of value to highlight some challenges that pilot studies or clinical trials might face when evaluating a radiological procedure similar to the Big 5—Jena eCS protocol. For example, it is recommended that every patient is evaluated with the same diagnosing imaging (computed tomography and echocardiography) and that the design of the study should strive to be prospective in nature, so that the diagnostic accuracy of the Big 5—Jena eCS protocol can be compared to cardiac ultrasound.
